# 

**Published:** 1825-05

**Authors:** 


### NOTICE TO CORRESPONDENTS.

*Those Gentlemen whose Papers were acknowledged last month, and have not appeared in the present Number, are requested to believe that the delay has been unavoidable. As it is, we have devoted more than the usual allowance of our pages to the "Original" department.*

Dr. VENABLE'S *Paper could not be inserted in this Number: it shall certainly appear in our next.*

Mr. GREEN'S *letter has been received, and his request shall be attended to.*

*Various Communications, which we have privately acknowledged, are preparing for publication.*

Mr. THOMAS'S *letter has been received; as have the Papers of* Dr. BAKER, Mr. CHEVALIER, *and* Mr. BLACKETT.

*Press of other matter obliges us to postpone the* List of Books *till next month.*

*N. B.—Regarding* Dr. THOMSON'S *account of his own case as extremely interesting*, we have added an additional quarter-sheet, that its insertion might not interfere with the other departments of the Journal.

